# Difficulties in facial emotion recognition: taking psychopathic and alexithymic traits into account

**DOI:** 10.1186/s40359-022-00946-x

**Published:** 2022-10-27

**Authors:** Melina Nicole Kyranides, Demetris Christofides, Melis Çetin

**Affiliations:** 1 Department of Clinical and Health Psychology, University of Edinburgh, Edinburgh, UK; 2 Mind Mental Health Care, London, UK

**Keywords:** Psychopathic traits, Alexithymic traits, Facial emotion recognition, Facial expressions

## Abstract

**Background:**

Alexithymic and psychopathic traits are closely associated, but distinct constructs. Both have been associated with facial emotion processing deficits reflecting empathy deficits, however the underlying mechanism contributing to these deficits is not well-understood.

**Methods:**

This study investigated facial emotion recognition performance in a non-clinical sample (*N* = 110) including both male and female participants, with varying levels of psychopathic and alexithymic traits. Facial emotion recognition was assessed using a computerized task, that presented different expressions depicting five emotional states (pain, fear, sadness, anger, happiness) and neutral expressions.

**Results:**

Results suggest that the high psychopathic traits group reported lower accuracy compared to the low psychopathic traits group, indicating a more generalized deficit in facial affect recognition, across all emotions. The alexithymic groups (high vs. low) on the other hand did not differ in their performance on the task for any of the emotions presented.

**Conclusion:**

These findings add to the current body of research regarding face processing categorization deficits in relation to psychopathic and alexithymic traits and can inform prevention and intervention efforts that aim to facilitate facial emotion recognition in individuals with these personality traits.

Nonverbal emotional expressions are central for developing and maintaining interpersonal relationships [1], because correctly identifying and categorizing facial expressions is one of the most common and efficient skills of nonverbal communication [2]. Successfully decoding facial emotional expressions and syncing into a similar affective state, are critical steps in experiencing empathy [3, 4]. However not all people are as skillful in processing subtle facial emotions, as is the case with individuals with psychopathic and/or alexithymic traits. Both alexithymic and psychopathic traits have been associated with emotional processing deficits resulting from facial emotion recognition difficulties [5, 6], and due to this, these traits have been associated with each other [7] yet the research in this area is limited and the findings controversial.

Psychopathy is a cluster of distinct personality characteristics such as callousness, lack of empathy, impulsivity and deceptiveness, which can be accompanied by socially unacceptable and criminal behavior [8, 9]. As a result, individuals with psychopathic traits show reduced empathy and guilt, as well as reduced attachment to significant others [10]. The prevalence rate among the general population is 1–2% and reaches 50% among violent offenders [11, 12]. Individuals with psychopathic traits have shallow emotional experiences, appear uninterested towards the feelings of others [5, 13] and have difficulties in facial emotion recognition [14, 15]. Alexithymic traits have been associated with difficulties in identifying and describing emotions, difficulties in differentiating between feelings and somatic sensations in the presence of emotional arousal, and preoccupation with externally-oriented thinking and deficits in empathy [3, 6, 16]. The prevalence rate of alexithymic traits when using a cut-off score of 61 on the Toronto Alexithymia Scale [16, 17] varies between 10 and 18% among non-clinical samples in different cultures [18, 19] but has also been reported in individuals on the autism spectrum [20] in individuals with depression [21] and in individuals with eating disorders [22].

There are strong conceptual similarities between the two constructs. Alexithymic traits, similarly to psychopathic traits have been often associated with interpersonal difficulties resulting from the lack of understanding of emotions [6], difficulties in forming and maintaining intimate emotional relationships [23, 24], both have been associated with insecure attachment [25, 26], problems in emotion regulation [25, 27] and have been associated with similar causal factors including childhood trauma [28, 29]. Both personality traits are considered highly costly to society due to their associated risk with aggression [27, 30, 31]. Despite these similarities, research has also revealed some differences. For example, individuals with alexithymic traits tend to experience high levels of anxiety, are submissive, unexciting, ethically consistent and socially conforming, while individuals with psychopathic traits usually experience low levels of anxiety, are dominant, charming, deceitful and nonconforming [32–34].

There are several studies exploring the relationship between psychopathic and alexithymic traits, with the findings being controversial. Some studies report a positive association between psychopathic and alexithymic traits [35, 36], others reported a negative association [7], while others report no such relationship [37]. Considering the common patterns in deficits in empathy and social functioning associated with these personality traits, it is important to examine how these deficits relate to facial emotion recognition more specifically. Understanding the overlap between these personality traits regarding deficits in facial emotion recognition will help identify some of the social functioning difficulties individuals with these traits face and shed some light on how to better manage them. This study aims to investigate how individuals with high and/or low alexithymic and psychopathic traits differ but also how they are similar, in correctly identifying facial expressions.

## Empathy and facial emotion recognition

Developing empathy (the capacity to understand and know the difference between one’s own emotions and feelings and those of another person) is important to maintain healthy social interactions, as it facilitates prosocial/altruistic behavior, while the absence of it has been linked to antisocial behavior and the presence of psychopathic and alexithymic traits [38, 39]. Empathy has different components; (1) an affective component needed for emotion recognition and emotional contagion (sharing that emotion, experiencing it), (2) a cognitive component of perspective taking needed for differentiating between the self and others, and (3) executive functions needed for combining different experiences of the perceiver to result in empathetic concern [40]. Motor mimicry and emotional contagion are the most basic expressions (precursors) of empathy and mature empathy (or highly skillful empathy) can be achieved, when both affective and cognitive processes are aligned, while empathetic behavior can be minimized when these are not aligned [41]. Emotional states of others are processed and synchronized through one’s own embodied representations and are dependent on psychological states and contextual factors [41]. According to the Self to Other Model of Empathy [3] individuals with psychopathic traits have difficulty in recognizing distressful facial emotions in others, compared to typically developing individuals, which prevents them from experiencing a matching state of emotion. This is critical because emotional contagion, which is the syncing of feelings experienced instinctively during interactions, is an important first step in experiencing empathy [3]. Observing and correctly identifying another person’s facial expressions can lead to sharing the subjective states between individuals and can be a reference for decoding the meaning of another person’s intentions.

## Psychopathic traits and facial emotion recognition

Research in individuals with psychopathic traits has shown that they have pronounced affective empathy deficits [41–43] which is thought to result from the fact that these individuals have a diminished capacity in experiencing emotions [12, 44, 45], at least distressing emotions (i.e., sadness, fear, possibly pain) with the majority of studies supporting the specific emotion deficit perspective. To be more precise, some studies on facial emotion recognition report a robust link between identifying sad and fearful affect recognition and psychopathic traits [42, 45, 46], but not for happiness, anger and disgust [47]. There is also evidence that these deficits extent to painful expressions among individuals with psychopathic traits [12, 48, 49]. Others have argued that there are deficits in both cognitive and affective empathy in individuals with psychopathic traits [35, 41] displaying a more generalized deficit for all emotions [14, 15, 42, 45] and that these deficits are largely attributed to deficits in general mental ability (e.g., [50]). Interestingly, a study that recruited a university sample, reported that individuals with high psychopathic traits were more accurate in recognizing fearful facial affect [51] while another study using a community sample reported significant deficits in fear recognition [52]. The aforementioned findings indicate the need for further research to clarify the link between psychopathic traits and the recognition of facial expressions in non-clinical samples, to identify if these deficits are unique to psychopathic traits or extent to other personality traits.

## Alexithymic traits and facial emotion recognition

At an interpersonal level, individuals with alexithymic traits also show poor empathy [53, 54] which results from their inability to construct a consciously available emotional state of themselves [3]. In other words, they notice that they are having an emotion, but they are not able to label the emotion they are experiencing. High alexithymic traits have been associated with reduced facial mimicry (emotional contagion) as this was recorded by significantly lower elecreomyographic activity on facial muscles in response to affective faces [55]. Hence, the emotion recognition deficit observed in individuals with alexithymic traits seems to occur early on in the information processing process, that affects all the following stages. Based on this it is expected that deficits are to be generalized rather than specific to distressing emotions as in the case of psychopathy [42, 45]. This is supported by a systematic review [56] that examined twelve studies, ten of which suggested that the deficits in the processing of emotional facial expressions in individuals with high alexithymic traits extended to all emotional categories (including anger, fear, sadness, happiness and neutral stimuli). High alexithymic traits have been linked with deficits in cognitive empathy [35, 57] and affective empathy [55, 58] while some studies [e.g., 58, 59] suggest that high alexithymic traits are associated predominantly with low cognitive empathy, while affective empathy is less affected, indicating more research is needed.

## The current study

Even though alexithymic and psychopathic traits have been both associated with facial emotion recognition deficits [6, 35], and empathy difficulties [3], no study to our knowledge has investigated the relationship between both alexithymic and psychopathic traits, with a focus on facial emotion recognition. Past research has examined these personality traits separately or has yielded inconsistent results regarding the relationship with facial emotion recognition deficits. Identifying common patterns between these two personality traits can shed light into similar underlying mechanisms contributing to these empathy deficits and maintenance of these personality traits but can also highlight the differences between the two, allowing us to better inform prevention and intervention efforts. Both personality traits are suggested to be associated with empathy related deficits, which are thought to result from different impairments during the empathy process [3]. While psychopathic traits are often related with deficits in predominantly affective empathy [40, 41], that is documented with a specific deficit in the recognition of distressed facial expressions, alexithymic traits show intact affective empathy [58, 59] but deficits with cognitive empathy [35, 57] that are presented as a generalized deficit for all emotions. Individuals with alexithymic traits seem to struggle to represent emotional states in themselves, that leads to deficits in correctly identifying emotions in others [56]. This might lead to a differentiation between alexithymic and psychopathic traits, since facial emotion recognition is considered to be an important skill that contributes to empathy [41]. This study expands previous research by looking at both personality traits in a nonclinical sample. This is important as studies have reported diverse expressions of these personality traits across the population [11, 19] and conducting research with individuals that are not institutionalized, allows for the wider generalizability of findings.

The current study aimed to investigate facial affect recognition in participants with varying levels of these personality traits. Participants were divided into groups with high or low psychopathic and high or low alexithymic traits, and examined their performance on a computerized task, assessing facial emotion recognition. It was hypothesized that individuals with high psychopathic traits and individuals with high alexithymic traits would perform worse on the facial emotion recognition task, compared to individuals with lower levels on these traits, reflecting their empathy deficits. Based on prior work [47, 49, 56], it was hypothesized that individuals with high psychopathic traits would show impaired recognition of fearful, painful and sad facial expressions more specifically, while a more generalized deficit in facial emotion recognition was hypothesized for individuals with higher alexithymic traits.

## Method

### Participants

The sample consisted of university students who were all over the age of 18 and fluent in English. An a priori power analysis using the G*Power software [60] indicated that a total sample of 108 participants would be needed to detect a small effect size with a power of 95% using a mixed ANOVA, testing main and interaction effects with alpha at 0.05. The total number of participants recruited for the study was 111 although the analysis was carried out with 110 (*M*age = 24.92, *SD* = 2.78) as one of the participant’s data was lost due to technical errors. The final sample consisted of 33 men and 77 women.

## Measures

***Youth Psychopathic Traits Inventory–Short version*** (YPI-S; [61]). The YPI-S includes 18 items from the original YPI [62] and assesses psychopathic traits. The measure comprises of three subscales: (a) the callous unemotional or affective dimension (6 items; e.g. *I think that crying is a sign of weakness even if no one sees you*), (b) the grandiose-manipulative or interpersonal dimension (6 items; e.g. *I have the ability to con people by using my charm and smile*) and (c) the impulsive-irresponsible or behavioural dimension (6 items; e.g. *It often happens that I do things without thinking ahead*). Items are rated on a four-point Likert scale, from 1 (“*Does not apply at all*”) to 4 (“*Applies very well*”). The YPI-S has shown a strong correlation (*r* = .95) with the original 50-item YPI and was found to have good construct validity and internal consistency [61]. The YPI-S has been used in prior studies with young adults [63]. In the current study the total score showed good internal consistency (*α* = 0.74).

***Toronto Alexithymia Scale*** (TAS-20; [16]). The TAS-20 includes 20-items and assesses the presence and severity of alexithymic traits. The TAS-20 includes three dimensions: (a) difficulty identifying and distinguishing between feelings and bodily sensations (7 items; e.g. *I am often puzzled by sensations in my body*), (b) difficulty describing feelings to others (5 items; e.g. *I find it hard to describe how I feel about people*), and (c) externally oriented thinking (8 items; e.g. *I prefer talking to people about their daily activities rather than their feelings*). Each item is rated on a 5-point Likert scale ranging from 1 (*strongly disagree*) to 5 (*strongly agree*). The scale has demonstrated good psychometric properties (*α* = 0.81) and convergent validity to observer-rated alexithymia [16]. In the present study, the total score showed good internal consistency (*α* = 0.80).

## Facial emotion recognition task

The featured stimuli used in the task were derived from the Montréal Pain and Affective Face Clips (MPAFC) database [64]. Dynamic facial expressions were chosen over static stimuli since they have greater ecological validity and have been used effectively in other studies [48]. The dynamic stimuli featured Ekman’s [65] basic emotions of happiness, sadness, anger, fear, pain in addition to a neutral faces. The 1-second clips featured trained actors that performed the emotional expressions. In total 48 stimuli were presented, with the six emotions being featured by four male and four female actors (6 × 8). Facial Action Coding System (FACS) confirms that the male and female expressions are comparable in frequency and intensity of the expression [66]. The task was set up in E-prime (E-prime 3.0) and was presented on laptops (screen dimensions: 40 × 30 cm). The facial expressions (dynamic stimuli) were displayed, in random order. Following the presentation of each facial expression, the stimuli would disappear and emotional labels would then appear on the screen. Participants were instructed to choose the number on the keyboard to select the corresponding emotional label that they believed, featured the emotional stimuli that was presented (i.e. 1 = sadness, 2 = happiness). Participants were asked to respond as fast and as accurately as they could.

## Procedure

The study was granted ethical approval from the University of Edinburgh ethics committee. All participants provided consent after reviewing all the relevant information. The experimental task was administered first followed by the questionnaires and the administration of measures was conducted at the University premises. The measures were administrated in the same order for all participants. The approximate duration of the computerized task was 10 min while the self-report measures took 15 min to complete. Participants were then debriefed and thanked for their time.

## Results

Data analysis was carried in SPSS version 25. In order to explore participants’ performance on the task, while taking into account alexithymic and psychopathic traits, the participants were divided into two groups based on the sample medians (high vs. low) on the TAS-20 which assessed alexithymic traits and the YPI-S which assessed psychopathic traits. The independent t-tests that were conducted to establish if the groups (high, low) differed based on their psychopathic and alexithymic traits, showed that the differences in psychopathic traits and alexithymic traits were significant (Table 1).

**Table 1:** Comparisons between identified groups for the study sample

Variable	Mean	SD	95% CI	T	Cohen’s d
*Psychopathic traits (YPI-S)*					
Low (*n* = 55)	27.71	3.66	(9.31 ; 12.10)	15.22*	2.90
High *(n* = 55)	38.42	3.73			
*Alexithymic traits (TAS-20)*					
Low (*n* = 55)	34.42	4.79	(12.84 ; 16.98)	14.27*	2.72
High (*n* = 55)	49.33	6.09			

*Note.* YPI-S: Youth Psychopathic Traits Inventory-Short version; TAS-20: Toronto Alexithymia Scale; SD: Standard Deviation; CI: Confidence Interval

*p < .001

## Accuracy

A mixed measures Analysis of Variance (ANOVA) was conducted with psychopathic traits group (high, low), alexithymic traits group (high, low), as the between-subjects variable and accuracy responses for the six facial expressions (anger, fear, sadness, pain, happiness, neutral) as the within-subjects variables. The 2 × 2 × 6 mixed ANOVA, revealed a significant group effect for the psychopathic traits group *F* (1, 106) = 6.82, *p* < .05, *η*²= 0.060. Bonferroni post-hoc comparisons showed that the high psychopathy group scored lower on overall accuracy (*M* = 88.35, *SE* = 0.84) compared to the control group with low psychopathic traits (*M* = 91.46 *SE* = 0.84). No main effect was identified for alexithymic group (*p =* .58) and no interaction was identified between the psychopathic and alexithymic groups (*p =* .90).

**Fig. 1:**
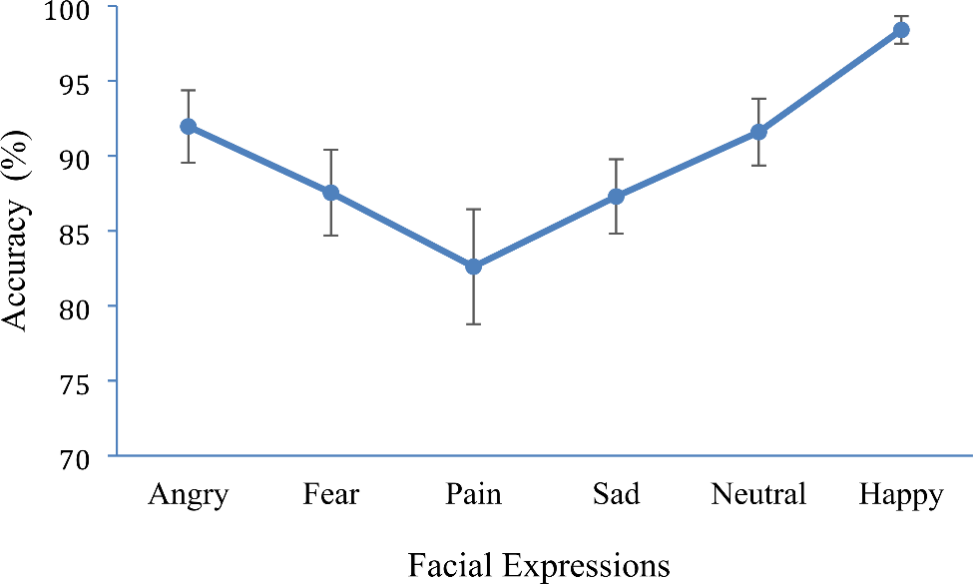
Accuracy rates for the different facial expressions. Note: Error bars show 95% confidence intervals

There was also a significant effect for emotion *F* (3.73, 395.39) = 17.65, *p* < .001, *η*²= 0.14. Participants were most accurate at identifying emotions in the following order: happy (*M* = 98.41, *SE* = 0.46), angry (*M* = 91.97, *SE* = 1.22), neutral (*M* = 91.60, *SE* = 1.13), fearful (*M* = 87.55, *SE* = 1.44), sad (*M* = 87.29, *SE* = 1.25) and lastly painful (*M* = 82.61, *SE* = 1.93) facial expressions (Fig. 1). Bonferroni post hoc comparisons indicated that participants were significantly more accurate at correctly identifying happy expressions compared to all other expressions (*ps* < 0.05). Painful facial expressions had significantly lower accuracy ratings compared to all other emotions except fearful and sad facial expressions (*p*s > 0.05). No other interactions were identified (*p*s > 0.05).

## Response time

**Fig. 2:**
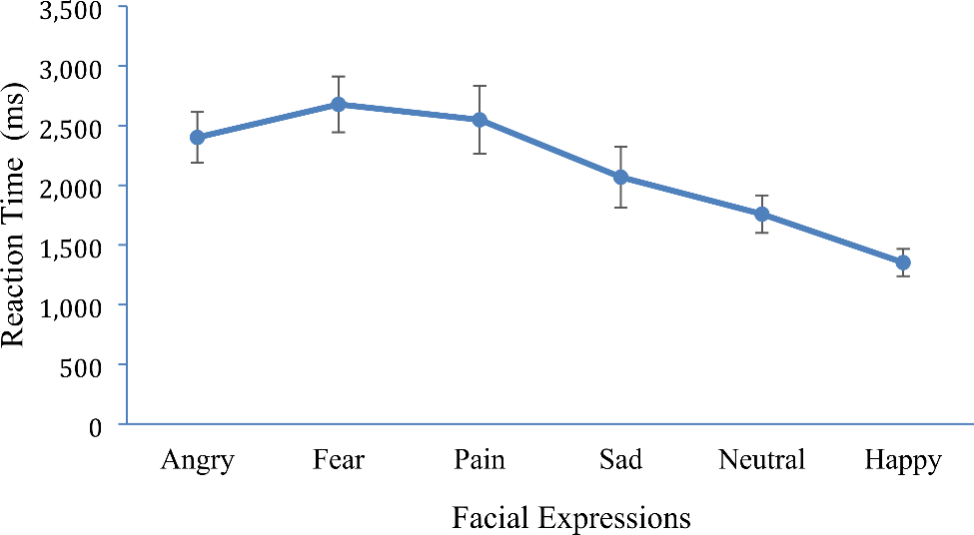
Reaction times for the different facial expressions. Note. Error bars show 95% confidence intervals

The mixed 2 × 2 × 6 ANOVA, for response times revealed no main effect for the psychopathic traits group (*p =* .45) or alexithymic group (*p =* .13) and no interaction between the psychopathic and alexithymic groups (*p =* .79). There was only a significant effect for emotion *F* (4.01, 424.45) = 28.59, *p* < .001, *η*²= 0.21. Participants were faster identifying emotions in the following order: happy (*M* = 1352.23, *SE* = 57.44), neutral (*M* = 1758.32, *SE* = 79.46), sad (*M* = 2068.04, *SE* = 128.65), angry (*M* = 2400.99, *SE* = 107.43), painful (*M* = 2548.57, *SE* = 143.47) and were slower for fearful (*M* = 2677.85, *SE* = 118.00) facial expressions (Fig. 2). Bonferroni post hoc comparisons indicated that participants were significantly faster at identifying happy expressions compared to all other expressions (*p*s < 0.05). Participants were faster at identifying neutral facial expressions compared to angry, fearful, painful facial expressions but not when compared to sad facial expressions. Participants were faster at identifying sad facial expressions compared to fearful but not angry or painful. No other differences in reaction times were significant. No other interactions were identified (*p*s > 0.05).

## Discussion

The aim of the present study was to investigate facial emotion recognition performance in individuals with different levels of psychopathic and alexithymic traits. It was hypothesized that the groups with higher levels of alexithymic and psychopathic traits would show worse performance on the task assessing facial affect recognition, reflecting deficits in empathy. The high psychopathic traits group was expected to perform worse in the recognition of distressing facial expressions (e.g., fearful, sad and painful) more specifically, while a more generalized deficit (across all emotions) was expected for individuals with high alexithymic traits. Findings revealed that individuals with higher psychopathic traits showed lower accuracy rates across all emotions not just distressing ones, compared to the control group (who were low in psychopathic traits) reflecting a more generalized deficit. This deficit applied to accuracy but not speed of emotional processing of facial expressions. Contrary to expectations, individuals with high alexithymic traits did not show any deficits, not when examining accuracy nor when examining response time.

The hypothesis regarding impairments in facial affect recognition for individuals with high psychopathic tendencies, was partially supported. Our findings suggest that individuals with psychopathic traits showed impairments in facial affect recognition but this was not specific to distressing emotions, but more generalized. Even though fear, sadness and pain recognition impairments in individuals with psychopathic traits has been documented in other studies [46, 47, 49] this was partially replicated in our study, with our findings suggesting a more extensive deficit across all emotions that has been supported by prior work [14, 15, 42, 43, 45]. The general emotional processing deficit [42, 45] implies an overall reduced capacity to process affective expressions across the emotional spectrum, while the specific emotional processing deficit [46, 47] only involves specific emotions (with a focus on distressing emotions, e.g., fear, sadness and pain). Empathizing with others in terms of their emotional feelings should be theoretically difficult if one is unable to recognize those emotions. If individuals with psychopathic traits fail to correctly identify and do not always comprehend others facial cues and their emotional state, they are more likely to engage with inappropriate behavior that negatively affects others [31, 32]. This might be related to a deficit in emotional contagion which is necessary for empathy to occur and develops over repeated pairing of affective states with cues of the other person over time [3]. It could also suggest deficits in both affective and cognitive empathy [41–43] or general mental ability (e.g., [50]), or reduced attention to face regions that results in difficulties deciphering facial expression [48, 67]. Unfortunately, we did not include a measure of general mental ability in our study to examine if this was the case. If these deficits are the result of lack of attention, this can be corrected at least temporarily, by directing participants’ attention to different face regions to help them notice important social cues and increase the possibility that they will correctly identify the facial expression [48, 68]. The possibility of influencing emotional processing in individuals with psychopathic traits might offer an avenue for future interventions efforts, although it is currently unknown whether improving emotion recognition will also lead to increasing empathy in the long term. The fact that we did not find selective deficits for distressing emotions to be related to psychopathic traits could be attributed to being unique to the situation/task used. Our study used dynamic facial stimuli, while other studies that reported specific deficits in fear, sadness and pain had used either static images of faces or morphed facial recognition tasks [46, 52]. It might be that the task used in the current study, while more ecologically valid, included subtle micro-movements that helped participants identify the facial expressions. Additionally we only included six emotional categories as options to choose from. Having a wider range of emotional categories would have allowed for more potential misattributions, something that can be explored in future studies.

Our study failed to detect a deficit (generalized or specific) for individuals with high alexithymic traits, in contrast to previous studies, which proposed a general difficulty in facial affect recognition [56, 69–71]. Our findings are aligned to studies that also failed to identify these deficits in facial affect recognition [72, 73], if emotional labels were provided. Providing emotional labels has been found to remove the emotion perception deficits for individuals with alexithymia [73]. It might be the case that the task became easier for the individuals with alexithymic traits in our study, as we provided emotional labels that appeared immediately after the stimuli (affective faces) disappeared from the screen. Accuracy rates might have been negatively affected for individuals with high alexithymic traits, if the task was modified to not include the labels and participants were asked to report the emotion [56]. Further to this, we did not assess verbal abilities to examine if these had an impact on the findings [74]. It might be that participants verbal abilities were intact and that might explain the lack of deficits in the group of individuals with high alexithymic traits. This should be examined in future work.

Performance on the task varied as a function of emotion category, which is aligned to previous studies documenting that happiness is an easy emotion to identify, as it obtained the highest accuracy scores, while pain, fear and sadness obtained the lowest scores, similar to prior work [48, 75, 76]. Response times also reflected a similar pattern of results suggesting slower reaction to negative emotions (fear, pain, angry sad) compared to positive emotions (happiness) which were identified faster [77].

The current findings should be interpreted in light of some limitations. Our sample consisted of young adults, the majority of which were female, attending university, so findings should be replicated in a clinical, gender balanced sample with individuals scoring higher on alexithymic and psychopathic traits. Psychopathic traits reported in the current study were similar to other studies using non-clinical sample of young adults [78] but were lower with regard to alexithymic traits reported in previous studies also using a community sample [71, 79] which might explain the lack of findings in relation to alexithymic traits. The face stimuli used in the current study were dynamic, reflecting facial expressions that are encountered in everyday life. However, the clips presented posed/enacted facial expressions as opposed to more naturalistic expressions of emotions elicited by particular situations that might be more subtle. Although posed/enacted facial expressions can be encountered in social situations, future research should seek to replicate the current findings using more spontaneous expressions from live interactions to further increase ecological validity. Additionally most of the target expressions in this study were negatively valenced (pain, sadness, fear, anger vs. happiness and neutral). Positive emotions are actively sought in daily life (e.g., [80]) but are also multifaceted so future research should extend our findings by considering a wider variety of positive facial expressions and their interactions with these personality traits. Future studies should also examine the relationship between these personality traits using distinct verbal and non-verbal tasks or control for participants’ verbal abilities. Despite these limitations, this study is the first to our knowledge to examine the influence of both alexithymic and psychopathic personality traits and facial emotion recognition deficits using an objective computerized task that included dynamic stimuli as oppose to static stimuli.

## Conclusion

The main focus of this study was to examine the dominant features of alexithymic and psychopathic traits in relation to facial affect recognition. Our study showed that individuals with high psychopathic traits performed worse on the facial emotion recognition task, producing more errors in comparison to those in the low-end group. Alexithymic traits on the other hand did not influence performance on the facial emotion recognition task. While both personality traits have been associated with empathy related difficulties, our results show that difficulties in facial emotion recognition are associated with psychopathic traits more predominantly. Future studies are encouraged to replicate these findings using a clinical sample that includes individuals with elevated levels of alexithymic and psychopathic traits. Training individuals to identify emotional cues through attending to facial features early on could foster empathy and pro-social behaviour [81] as emotion recognition is a subcomponent of empathy. Increasing the ability to correctly identify facial expressions, can be achieved by directing participants’ attention to facial features [48] and by encouraging emotional contagion [67]. These studies suggest that we can improve facial affect recognition which can hopefully improve interpersonal relationships for individuals with psychopathic traits.

## Data Availability

The datasets generated during and/or analyzed during the current study are available from the corresponding author on reasonable request.
